# Macroscale White Matter Alterations Due to Traumatic Cerebral Microhemorrhages Are Revealed by Diffusion Tensor Imaging

**DOI:** 10.3389/fneur.2018.00948

**Published:** 2018-11-13

**Authors:** Kenneth A. Rostowsky, Alexander S. Maher, Andrei Irimia

**Affiliations:** Ethel Percy Andrus Gerontology Center, USC Leonard Davis School of Gerontology, University of Southern California, Los Angeles, CA, United States

**Keywords:** traumatic brain injury, cerebral microbleed, diffusion tensor imaging, connectomics, susceptibility weighted imaging

## Abstract

With the advent of susceptibility-weighted imaging (SWI), the ability to identify cerebral microbleeds (CMBs) associated with mild traumatic brain injury (mTBI) has become increasingly commonplace. Nevertheless, the clinical significance of post-traumatic CMBs remains controversial partly because it is unclear whether mTBI-related CMBs entail brain circuitry disruptions which, although structurally subtle, are functionally significant. This study combines magnetic resonance and diffusion tensor imaging (MRI and DTI) to map white matter (WM) circuitry differences across 6 months in 26 healthy control volunteers and in 26 older mTBI victims with acute CMBs of traumatic etiology. Six months post-mTBI, significant changes (*p* < 0.001) in the mean fractional anisotropy of perilesional WM bundles were identified in 21 volunteers, and an average of 47% (σ = 21%) of TBI-related CMBs were associated with such changes. These results suggest that CMBs can be associated with lasting changes in perilesional WM properties, even relatively far from CMB locations. Future strategies for mTBI care will likely rely on the ability to assess how subtle circuitry changes impact neural/cognitive function. Thus, assessing CMB effects upon the structural connectome can play a useful role when studying CMB sequelae and their potential impact upon the clinical outcome of individuals with concussion.

## Introduction

Using magnetic resonance, diffusion-weighted and diffusion tensor imaging (MRI, DWI and DTI, respectively) to quantify within-subject changes in white matter (WM) properties across time remains a particularly challenging task of brain image analysis (1). Specifically, to identify true longitudinal WM changes in the context of patient-tailored analysis, the neuroimaging researcher must address major challenges associated with the inherent technical limitations of diffusion imaging. For example, even when using the same acquisition protocol at each time point when MRI volumes are acquired, substantial artifactual differences between scans can often be present across imaging volumes acquired from the same subject. Such differences may pertain to (A) the signal-to-noise ratio (SNR) of MRI/DWI measurements, (B) the magnitude and spatial distribution of magnetic susceptibility artifacts, (C) the spatial pattern and extent of subject motion during data acquisition, etc. These and other confounds can lead to inaccurate calculation of diffusion parameters, to imprecise estimation of tensors for DTI analysis, and to subsequent loss of reliability when making scientific inferences. In studies of traumatic brain injury (TBI), the magnitude of such confounds can be even larger than in other populations due to factors such as (A) patients' poor ability to control their head motion while in the MRI scanner, (B) substantial injury-related variations in magnetic susceptibility throughout the brain, (C) the potential inability to calculate diffusion tensor parameters in hemorrhagic/edematous brain regions, etc. Thus, although potentially very useful, longitudinal analysis of WM changes prompted by TBI remains particularly challenging.

When applied to conditions like TBI, stroke or multiple sclerosis (MS), DTI approaches for identifying patterns of brain connectivity change at the population level can often be inadequate due to the large degree of inter-subject variability encountered in these clinical conditions. In fact, the accuracy of standard image processing operations—such as the co-registration of DWI/DTI volumes with structural MRI volumes—cannot be guaranteed to be successful even in healthy adults, particularly from the standpoint of resolving topological variabilities and from the perspective of aligning very fine neuroanatomical structures (2). Indeed, difficulties related to the reliable, within-subject co-registration of MRI/DWI volumes can greatly complicate the task of their longitudinal analysis. Although considerable effort has been dedicated to the task of alleviating image processing errors during *population-level* analysis of DWI/DTI datasets, addressing this problem when undertaking longitudinal, *subject-level* studies has received substantially less attention. Nevertheless, the need of reliable strategies for this purpose remains strong, particularly when considering the relevance of such methods to the analysis of neurological conditions associated with (A) high inter-patient variabilities in brain structure, (B) substantial heterogeneity in observed neuroanatomical deviations from normality, and (C) structurally subtle—yet functionally-significant—longitudinal changes.

The first aim of this study is to illustrate a state-of-the-art, patient-tailored method for the longitudinal analysis of WM circuitry and for the quantitation of brain connectivity changes due to hemorrhagic brain lesions. The second aim is to use this approach to investigate whether TBI-related cerebral microbleeds (CMBs) can be associated with long-range WM connectivity disruptions which are measurable using DWI and quantifiable via DTI tractography and related methods. The technique is applied to two longitudinal datasets, both consisting of repeated DWI/DTI measurements acquired from (A) 26 healthy adults and (B) 26 victims of geriatric mild TBI (mTBI). The mTBI cohort includes older volunteers whose only prominent, MRI-detectable form of brain pathology consists of mTBI-related CMBs. Part of our motivation for focusing on geriatric mTBI is the fact that, although CMBs have been identified frequently in the brains of TBI survivors (3), they are relatively less common in younger victims (4). Furthermore, the relationship between TBI-related CMBs and neurodegenerative disorders is of interest (5). Based on their acute presentation on multimodal MRI scans and on their longitudinal evolution, the CMBs studied here can be distinguished from microhemorrhages of chronic etiology, such as hypertensive vasculopathy or chronic amyloid angiopathy (6). Partly because the neuroanatomic changes effected by post-traumatic CMB evolution are typically subtler than those of larger, MRI-detectable lesions (7), CMB-related mass effects (e.g., compression, displacement) upon perilesional WM are presumably more moderate than those associated with larger lesions. For this reason, we argue that illustrating our technical approach by quantifying the structural effects of CMBs upon WM connectivity likely imposes a relatively-stringent and rigorous illustration of our method's robustness and effectiveness. Based on the results of our analysis, we argue that mTBI-related CMBs can be associated with disruptions of WM connectivity and that such disruptions may even affect brain connections which extend relatively far from the CMBs' immediate spatial neighborhoods. Importantly, our insights highlight the scientific and translational significance of patient-tailored DWI/DTI analysis and provide compelling arguments in favor of implementing rigorous approaches to the patient-tailored study of WM integrity and connectomics in neurological conditions where subtle-to-severe neuroanatomic abnormalities may be present. Finally, our contribution indicates that such methods—though often requiring sophisticated and ingenious analytics—should be automated solutions which are accessible to experts and novices alike without time-consuming user intervention.

## Materials and methods

### Participants

This study was carried out in accordance with the recommendations of the Code of Federal Regulations (45 C.F.R. 46) of the US Federal Government. The protocol was approved by the Institutional Review Board (IRB) at the University of Southern California. All subjects gave written informed consent in accordance with the Declaration of Helsinki. Participant demographics are summarized in Table 1. The two groups of primary interest to our study consisted of *N*_1_ = 26 (13 females) mTBI victims and *N*_2_ = 26 (13 females) healthy control (HC) volunteers. The Glasgow Coma Scale (GCS) score upon hospital emergency room (ER) admission was available for each mTBI volunteer (mean μ = 13.7; standard deviation σ = 0.4). Injury etiology involved falls due to loss of balance either while walking (22 subjects), riding a bicycle (2 subjects), running (1 subject) or playing a contact sport (1 subject). Volunteers in the HC and mTBI groups were matched according to their sexes and ages (HC: μ = 67.2 years, σ = 5.6 years; mTBI: μ = 66.8 years, σ = 5.9 years). The statistical significance of age and GCS score differences between the two cohorts was evaluated using Welch's *t*-test for samples with unequal variances, and the appropriate number of degrees of freedom were calculated according to the Welch-Satterthwaite approximation. A smaller, third group consisting of *N*_0_ = 6 young healthy control (HC) adults (three females of age 22, 28, and 32, respectively; three males of age 24, 32, and 35, respectively) were also included in the study. This was done to estimate the variance of our quantitative DTI metrics due to the cumulative effects of potential confounds such as measurement noise, motion artifacts, etc.

**Table 1:** Sample demographics for mTBI and both old and young HC participants.

**Cohort**	***N***	**Age [years]**	**Sex ratio**	**CMB load**	**GCS**
mTBI	26	66.8 ± 5.93	1:1	6.04 ± 2.63	13.7 ± 0.4
Old HC	26	67.2 ± 5.62	1:1	0.00 ± 0.00	—
Young HC	4	28.8 ± 5.08	1:1	0.00 ± 0.00	—

*CMB load refers to the number of CMBs identified via GRE/SWI. Averages and standard deviations are reported. GCS scores are unavailable for HC participants because these volunteers had not suffered any head injury at the time of the study*.

### Recruitment and inclusion/exclusion criteria

Volunteers were recruited through dissemination of IRB-approved material to identify prospective study participants. Potential enrollees were screened by a research-reliable investigator both by telephone and in person to ensure that inclusion/exclusion criteria were satisfied. Aside from the *N*_0_ = 6 young adults, only individuals aged 65 or older were included in the study because TBI-related CMBs are most likely to be present in the brains of older individuals (7), such that this is a particularly convenient population from which to sample. Only volunteers who had a GCS score equal to or greater than 13 upon ER admission were included in the mTBI group. Volunteers were excluded if they had a history of neurological disorder or psychiatric disease prior to the study. Participants with mTBI were excluded if they had a history of head trauma other than the most recent mTBI which qualified them for the study. Only TBI victims with CMBs were included; no volunteers were included if their acute neuroimaging scans indicated the presence of other types of TBI-related structural brain pathology. Furthermore, TBI volunteers were included only if their SWI-resolvable CMBs could be confidently linked to their TBI rather than to chronic conditions such as hypertensive vasculopathy or CAA. To achieve this distinction, CMBs were classified as TBI-related based on the criteria described in the *Lesion Identification* subsection.

### Neuroimaging

For the young HC volunteers (*N*_0_ = 6) included in the study for validation purposes described farther below, a total of four scanning sessions were held approximately 1 week apart. For mTBI volunteers, imaging sessions were held both acutely and chronically (i.e., 3 days or fewer post-TBI and again ~6 months after injury, respectively). For older HC volunteers, imaging sessions were held about 6 months apart, subject to the inclusion criterion that all volunteers had to be 65 years old or older by the time of the initial imaging session. MRI [including *T*_1_- and *T*_2_-weighted volumes, fluid-attenuated inversion recovery (FLAIR), gradient recalled echo (GRE)/susceptibility weighted imaging (SWI)] and gradient-echo (GE) DWI volumes were acquired using the same MRI scanner type (Prisma MAGNETOM Trio TIM with a 20-channel head coil, Siemens Corporation, Erlangen, Germany) and at the same magnetic field strength (3 T). *T*_1_-weighted imaging volumes of the head were acquired using a three-dimensional (3D), magnetization-prepared rapid acquisition gradient echo (MP-RAGE) sequence with the following acquisition parameters: repetition time (*T*_*R*_) = 1,950 ms; echo time (*T*_*E*_) = 2.98 ms; inversion time (*T*_*I*_) = 900 ms; echo train length (ETL) = 1; flip angle = 9 degrees; field of view (FOV) = 256 mm × 256 mm; matrix size = 256 × 256; slice thickness = 1 mm; slice oversaturation = 45.5%; sampling = 100%; phase encoding direction: anterior to posterior; acquisition bandwidth (BW) = 240 Hz; echo spacing = 6.8 ms. *T*_2_-weighted volumes were acquired using a 3D sequence (*T*_*R*_ = 2,500 ms; *T*_*E*_ = 360 ms; flip angle = 120 degrees; ETL = 180; FOV = 256 mm × 256 mm; matrix size = 256 × 256; slice thickness = 1 mm; phase encoding direction: anterior to posterior; BW = 750 Hz; echo spacing = 3.16 ms; Turbo factor = 141). FLAIR volumes were acquired axially (*T*_*R*_ = 9,000 ms; *T*_*E*_ = 78 ms; *T*_*I*_ = 2,500 ms; ETL = 15; flip angle = 150 degrees; FOV = 256 mm × 192 mm; matrix size = 256 × 192; slice thickness = 2 mm; sampling = 100%; phase encoding direction: anterior to posterior; BW = 250 Hz). Flow-compensated GRE/SWI volumes were acquired axially (*T*_*R*_ = 30 ms; *T*_*E*_ = 20 ms; FOV = 256 mm × 192 mm; matrix size = 512 × 256; slice thickness = 2 mm; phase encoding direction: right to left; BW = 100 Hz). DWI volumes were acquired axially in 64 gradient directions (*T*_*R*_ = 8,300 ms; *T*_*E*_ = 72 ms; flip angle = 90 degrees; ETL = 47; FOV = 256 mm × 256 mm; acquisition matrix size = 128 × 128; slice thickness = 2 mm; percentage sampling = 100; phase encoding direction: anterior to posterior; BW = 1,345 Hz; echo spacing = 0.83 ms; Turbo factor = 128). One volume with *b* = 0 s/mm^2^ and another with *b* = 1,000 s/mm^2^ (where *b* is the diffusion-weighting constant of DWI) were also acquired. All neuroimaging data were de-identified and de-linked after their acquisition.

### Pre-processing

**Figure 1:**
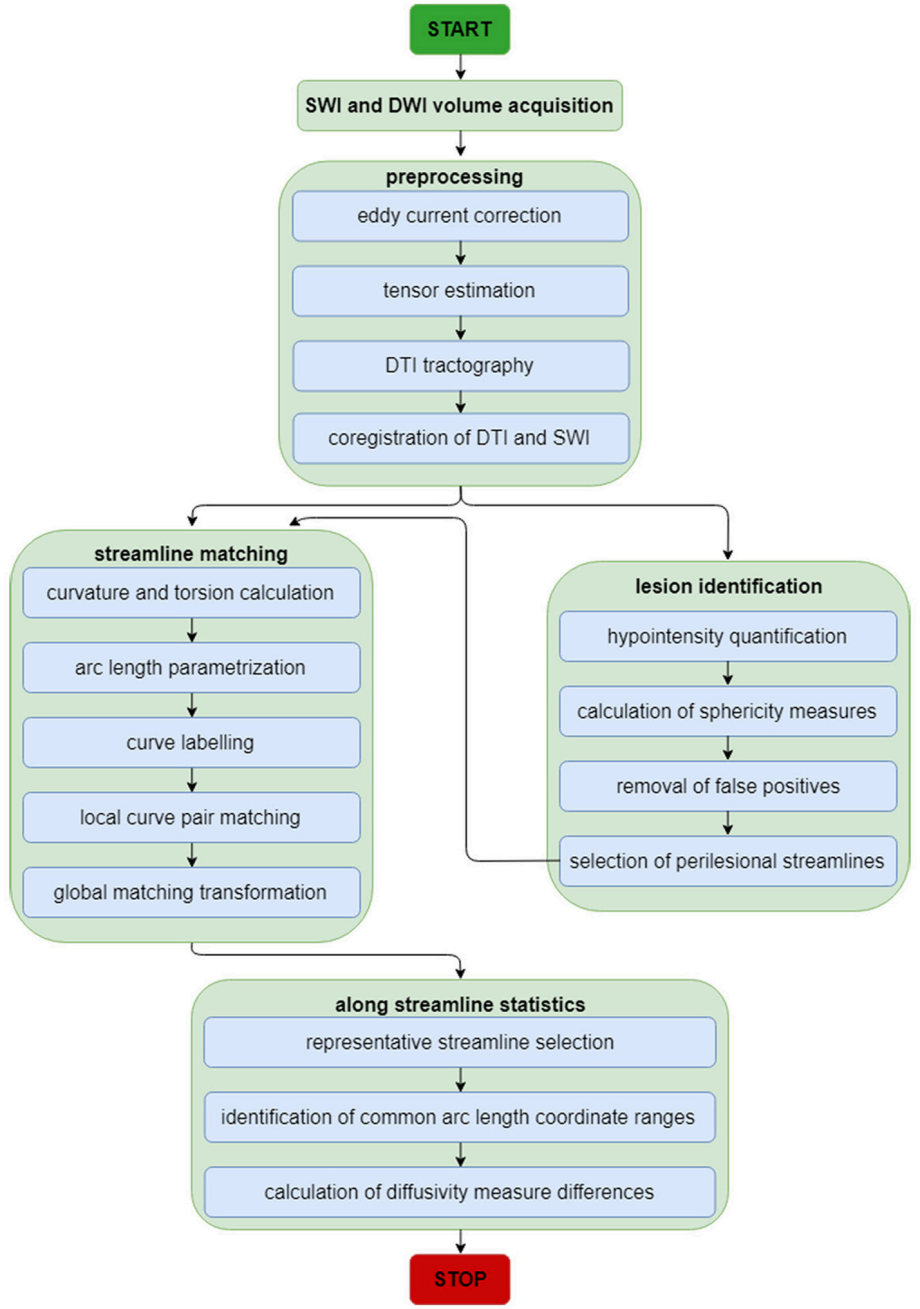
Neuroimage analysis flowchart for SWI and DWI volumes. The flowchart summarizes the analytic steps described in the *Methods* section, to which the reader is referred for details. Briefly, after standard pre-processing of SWI and DWI volumes, DTI streamlines and SWI volumes are each used for streamline matching and lesion identification, respectively. After the latter operation, the parameters of identified lesions are used as additional input to the streamline matching process. Once streamlines have been matched, statistics of interest are computed along streamlines.

For this and subsequent subsections, the reader is referred to Figure 1 for a schematic representation of the image analysis workflow, which is also summarized elsewhere (8). First, as a preprocessing step, imaging volumes were skull-stripped and their intensities were normalized across volunteers to reduce the potential confound of intensity differences across subjects when performing lesion identification. Intensity normalization was implemented by dividing the intensity values associated with voxels within the brain mask by the median intensity of these voxels. Eddy-current corrections were applied to each DWI volume, followed by an appropriate rotation of the **B** matrix (9). Bias field correction was implemented using the algorithm of Sled et al. (10) because this approach has been shown to be adequate when processing GRE/SWI volumes (11). In the study cohort, time-dependent changes in neuroanatomy are likely due primarily to traumatic axonal injury (TAI) and to CMB-related changes because (A) only volunteers whose MRI scans exhibited these types of pathology were included in the study, and because (B) no CMB was observed to appear/disappear across time points in any volunteers. In the HC cohort, where there were no incidental findings, observed neuroanatomic differences are likely due mostly to natural brain aging. Consequently, for each study volunteer, changes within each set of longitudinally-acquired MRI/DWI volumes were assumed to be diffeomorphic (one-to-one). For this reason, the skull-stripped brains—as acquired using each imaging modality—were co-registered to the *T*_1_-weighted volume using a rigid, affine, six-parameter registration.

### Lesion identification

A voxel classifier was used to identify image features as CMB candidates using the Microbleed Anatomic Rating Scale (MARS) guidelines (12), which classify CMB labels as either certain or doubtful. The former type of CMB is defined as a small, spherical or circular, clearly-defined hypo-intensity which features clear margins within the brain parenchyma. The latter type of CMB deviates from sphericity or circularity and/or is less well defined, i.e., more isointense than a definite CMB. Hypo-intensities connected to the boundary of the brain are not marked as CMBs because, by definition, CMBs are not connected to the meninges. False positive detections were removed using a second, object-based classifier which took into consideration the shapes of CMB candidates before finally labeling candidates as CMBs. CMB deviation from sphericity was quantified using the algorithm of ter Haar Romeny (13), which calculates the sphericity of a CMB from its curvature. To estimate the sensitivity and specificity of the lesion identification procedure, the results of the automated algorithm were compared to the manual labels assigned by a human expert with training in neuroanatomy and neuroradiology. SWI-resolvable CMBs were retained for subsequent analysis only if their etiology could be linked confidently to TBI rather than to chronic conditions such as hypertensive vasculopathy and CAA, both of which are common in the elderly (14). Specifically, each CMB was assumed to be TBI-related only if the following two conditions were satisfied:

(1) At the acute stage of injury, the CMB had to be surrounded—either partially or entirely—by one or several focal FLAIR hyperintensities which were fully adjacent to the CMB in question and whose contour encircled that the CMB;
(2) By the chronic stage of injury, the said FLAIR hyperintensities had to be undetectable on FLAIR scans.

If both conditions above were satisfied, the CMB was assumed to be TBI-related because (A) the acute post-TBI presence of peri-hemorrhagic FLAIR hyperintensities is very frequently indicative of post-traumatic edema (15), and (B) the absence of acutely-detected CMBs on chronic FLAIR scans is consistent with the resolution of such edema post-TBI (3). By contrast, in conditions such as hypertensive vasculopathy or CAA, the voxel intensity of chronic edema surrounding CMBs on FLAIR MRI typically changes very little over the course of 6 months (16, 17). For these reasons, the two criteria listed above are likely necessary and sufficient to allow CMB etiology to be established from multimodal, longitudinal SWI and FLAIR scans.

### DTI tractography

DWI volumes were processed in TrackVis (http://trackvis.org) and 3D Slicer (http://www.slicer.org). Tensors were fit to DWI data to perform DTI, and tractography streamlines were reconstructed using deterministic tractography subject to the following parameters: seed spacing = 0.5 mm; linear measure start threshold = 0.3; stopping value = 0.17; minimum streamline length = 20 mm; maximum streamline length = 110 mm; stopping criterion = fractional anisotropy (FA) value; stopping criterion value = 0.17; stopping track curvature = 0.96; integration step length = 0.5 mm. The algorithm used for tractography was fixed step-length streamline propagation. Here and throughout, the term “streamline bundles” is used when referring to the DTI-resolved proxies of WM tracts, in order to emphasize that WM fibers' tractography streamline counts should not be expected to equate to axonal counts (18).

### DTI measurement and tractography confounds

Before assessing time-related changes in DTI-resolved WM streamline properties, it is both prudent and useful to estimate the extent to which tractography measures can differ due to confounds (e.g., scanner noise, subject motion, magnetic field inhomogeneity differences, tensor estimation inaccuracies, tractography reconstruction errors, etc.) rather than to actual WM changes effected by the biological processes being studied, such as injury progression/resolution. The cumulative effects of confounds can be quantified by comparing repeated measurements acquired from HC subjects across relatively-short time intervals on the order of days. Specifically, if the MR acquisition parameters and DTI tractography/analysis workflows are identical across MRI acquisition sessions, this type of analysis can allow one to estimate the extent to which perceived WM differences are due to confounds rather than to the biological phenomena under investigation. This is the motivation behind our decision to include *N*_0_ = 6 young HC subjects, from whom MRI data were acquired over four sessions held at 1-week intervals. Thanks to the availability of these data, the cumulative effects of confounds upon DTI metrics of interest can be estimated and a useful baseline can be established to provide a reference distribution of metric values against which CMB-related effects upon brain circuitry can be compared.

Confound effects upon tractography metrics can be quantified by estimating, at each WM voxel, the extent of that voxel's spatial neighborhood within which any DTI-derived differences in WM properties being measured across time are likely due to artifacts rather than to TBI-related phenomena visible on MRI scans. Conceptually, the major axis of an ellipsoid centered at a voxel's location is a useful parameter to describe the extent of the spatial neighborhood in question. In loose analogy with the confidence (hyper-)ellipsoid of multivariate statistical analysis (19), we name this neighborhood the *uncertainty ellipsoid* of the voxel and its major axis the *uncertainty radius*. The uncertainty ellipsoid can be conceptualized as the spatial region around a voxel within which any perceived between-scan differences in DTI-derived WM measures should be interpreted with great caution due to their high likelihood to be confounded by measurement noise, motion artifacts, DTI tractography errors, etc. One advantage of quantifying such uncertainty using an ellipsoid rather than a sphere is the usefulness of accounting for the local anisotropy of water diffusion in the WM. As described in subsequent subsections, an approach to overcoming artifact-related confounds involves the implementation of methods like DTI streamline matching and prototyping, which allow one to focus on large-scale, topologically-consistent WM changes, i.e., on DTI streamlines whose properties differ substantially both across time points *and* over a spatial extent which is sufficiently larger than the uncertainty ellipsoid. When used together, these techniques can facilitate estimation of the mean value and standard deviation of the uncertainty radius within the WM. Here we restrict this estimation to the corpus callosum because (A) the trajectories of its WM fibers have been mapped extensively (20), (2) its inter-subject variability has been well quantified (21), (3) the WM tracts belonging to this structure are relatively straightforward to reconstruct from DTI tractography, and (4) callosal WM fibers have relatively well-behaved geometries, thereby reducing the risk of their resolvability being poor—as in the case of kissing or crossing DTI streamlines) (22, 23).

### DTI streamline matching

**Figure 2:**
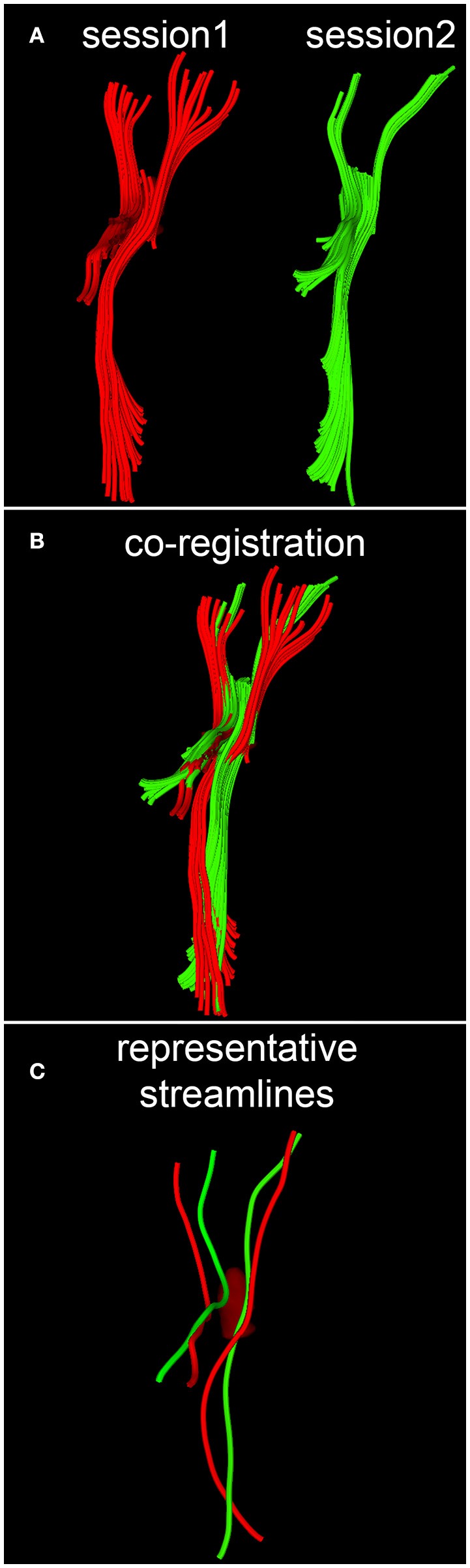
Conceptual representation of streamline matching, which involves **(A)** the reconstruction of DTI streamlines from DWI volumes acquired during each session, **(B)** the co-registration of streamlines within each subject, and **(C)** the selection of a streamline which is representative of a bundle's spatial trajectory.

When quantifying within-subject WM circuitry differences, it is important to address the task of matching DTI tractography streamlines across time points. Given that some streamlines and/or their reconstructed trajectories may be artifactual for reasons described previously as well as for a host of other reasons, we argue that this analysis step is necessary if realistic estimates of longitudinal WM differences are to be obtained. In what follows, the assumption is made that streamlines can be treated as piecewise-differentiable three-dimensional (3D) space curves with rotationally- and translationally-invariant curvature κ and torsion τ. Calculation of these two properties allows direct labeling of corresponding space curves to be established, local transformations of curve pairs to be calculated, and a final global transformation to be estimated, as described by Leemans et al. (24). Briefly, the intrinsic properties of any parametrized, regular space curve are uniquely defined by its curvature and torsion. For this reason, as described by differential geometry (25), any two curves which lie within a rigid transformation of each other are guaranteed to have identical curvature and torsion, provided that a one-to-one correspondence (called curve index correspondence) exists between parametrizations. To establish curve index correspondence, the appealing tract-based morphometry (TBM) approach of O'Donnell and Westin (26) is used in this study. This approach involves using subject-specific tractography bundle segmentations to generate arc length parametrizations of each bundle with point correspondences across all time points, thereby allowing tract-based measurement and analysis. Once curve index correspondence has been established within each subject, for each space curve and across all time points, a local transformation is computed to implement point-to-point co-registration via Schönemann's solution to the orthogonal Procrustes problem (24). Next, a global transformation which maps source curves to target curves is estimated from the set of local curve transformations by minimizing the global residue of squared inter-curve distances. A conceptual, pictorial representation of the DTI streamline matching process is shown in Figure 2, where DTI tractography streamlines are reconstructed based on DWI volumes acquired at different time points (Figure 2A), and the reconstructed streamlines are then co-registered within each subject (Figure 2B).

### Along-tract analysis

Once curves have been matched, the distance map method of Maddah et al. (27) and the streamline affinity approach of O'Donnell and Westin (26) can be used *in tandem* to perform streamline prototyping. This procedure involves the selection of a streamline which is most representative of a bundle's spatial trajectory (Figure 2C). As in the approach of O'Donnell and Westin (26), along-streamline measures of diffusivity (e.g., FA) can be calculated for each streamline's arc-length coordinate, and the descriptive statistics of each measure (mean, variance) are evaluated point wise at each of these coordinates. Because the streamlines within each bundle often vary in length, a curve portion amenable to the calculation of along-tract statistics must be identified. In this study, the curve portion in question is chosen as the range of arc-length coordinates which are common to a preponderance of streamlines in a bundle of interest. To illustrate along-tract analysis, we attempted to identify between-scan differences in the mean FA of perilesional streamlines. The mean FA was chosen because it is a widely-used DTI measure which depends on all three eigenvalues of the diffusion tensor. To determine whether between-scan differences in the mean FA of perilesional streamline bundles were unlikely to be artifactual, these differences had to be compared against empirically-established reference distributions of mean FA differences between scans. The mean and variance of these reference distributions were first calculated over streamlines which had been matched across scans, and two reference distributions were determined, one for each younger HC volunteer and another for each older HC volunteer. The distribution parameter values obtained within each of these subjects were then pooled within their own group (i.e., once over all younger HC volunteers and another time over all older HC volunteers). For each perilesional streamline bundle of interest, the null hypothesis was formulated as the statement that there was no between-session difference in the mean FA of streamlines within the bundle in question. This hypothesis was tested within the formalism of an analysis of variance (ANOVA) where sex was treated as a covariate whereas age group (young vs. old) and diagnosis (HC vs. mTBI) were used for stratification and comparison. The statistical significance of each hypothesis test was calculated at the α = 0.05 level, subject to the family-wise error rate (FWER) correction for multiple comparisons (28).

## Results

### Demographics and pathology findings

As expected, the mean age difference between groups was not significant (Welch's *t*_23.93_ = −0.78, *p* > 0.77). By design, there is no sex ratio difference across groups. TBI-related CMBs were first identified visually and labeled by a human rater trained in their identification, and the process was then repeated using the automated method. The average sensitivity of the automatic algorithm was found to be 94.4% (σ = 4.2%) across all volunteers. Thus, for this particular dataset, the classification accuracy is comparable or superior to that achieved by Bian et al. (29), Ghafaryasl et al. (30), and by van den Heuvel et al. (11) in similar studies. TBI-related CMBs were identified in the brain of each mTBI volunteer, with no preferential localization to any specific region. Across mTBI volunteers, the count of TBI-related CMBs ranged from 2 to 13 (μ = 6.04, σ = 2.6). No CMBs were identified in any of the HC volunteers, whether younger or older. The correlative relationships between CMB count, age, sex, and other demographic variables are not reported here because our sample sizes, while adequate for a methodological study such as ours, are undesirably small for this type of statistical analysis.

### Alleviation of DTI tractography confounds

**Figure 3:**
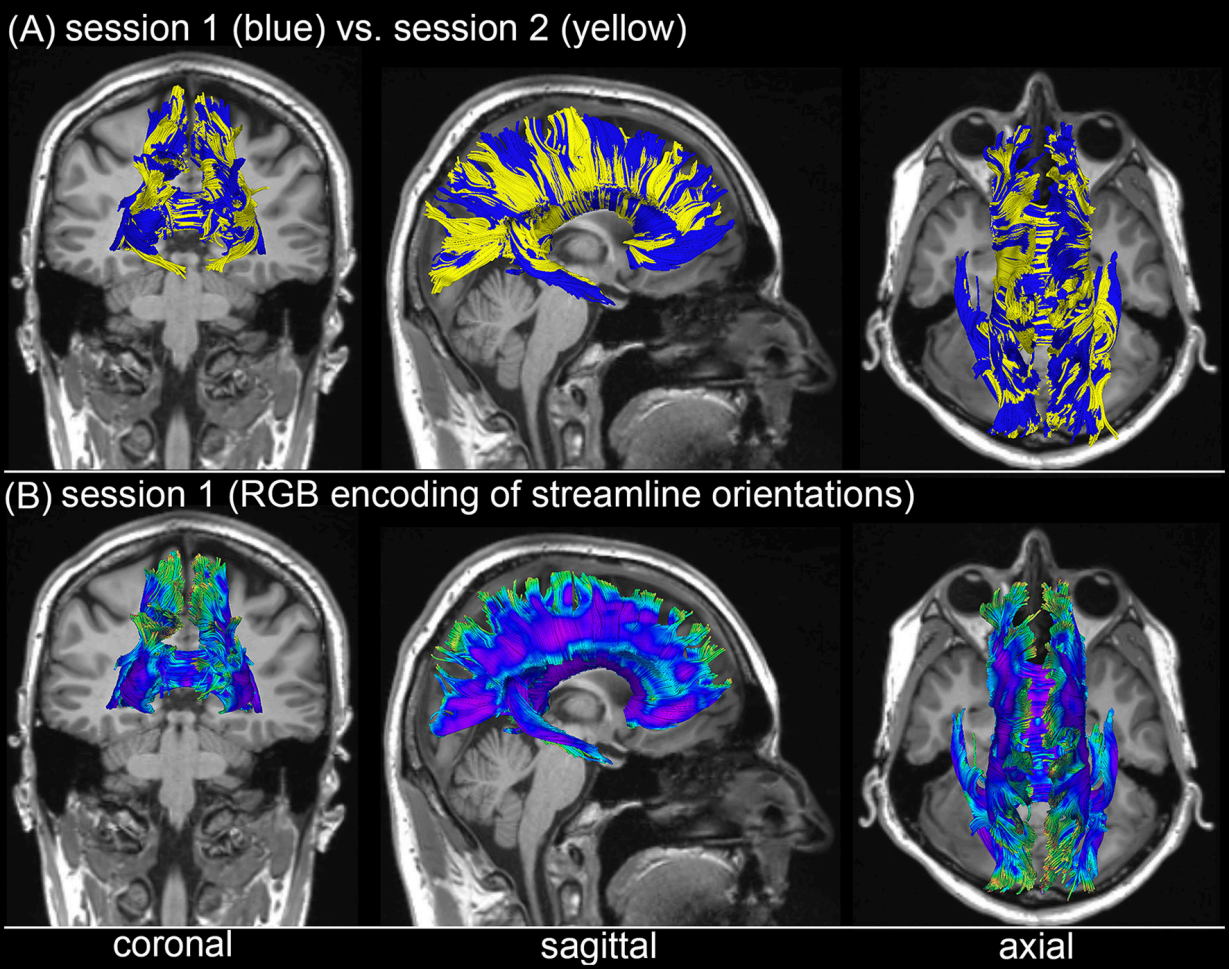
Depiction of DTI-derived streamlines in the corpus callosum superimposed on the *T*_1_-weighted MRI scans of a sample HC volunteer. **(A)** Two sets of streamlines (blue, yellow) were reconstructed based on scans acquired during distinct sessions. Despite similarities between the two sets of streamline bundles, noticeable differences abound. **(B)** When displayed using red-green-blue (RGB) encoding of streamline orientations, the DTI streamlines derived from scans acquired during session 1 are consistent with the expected neuroanatomy of the healthy corpus callosum.

**Figure 4:**
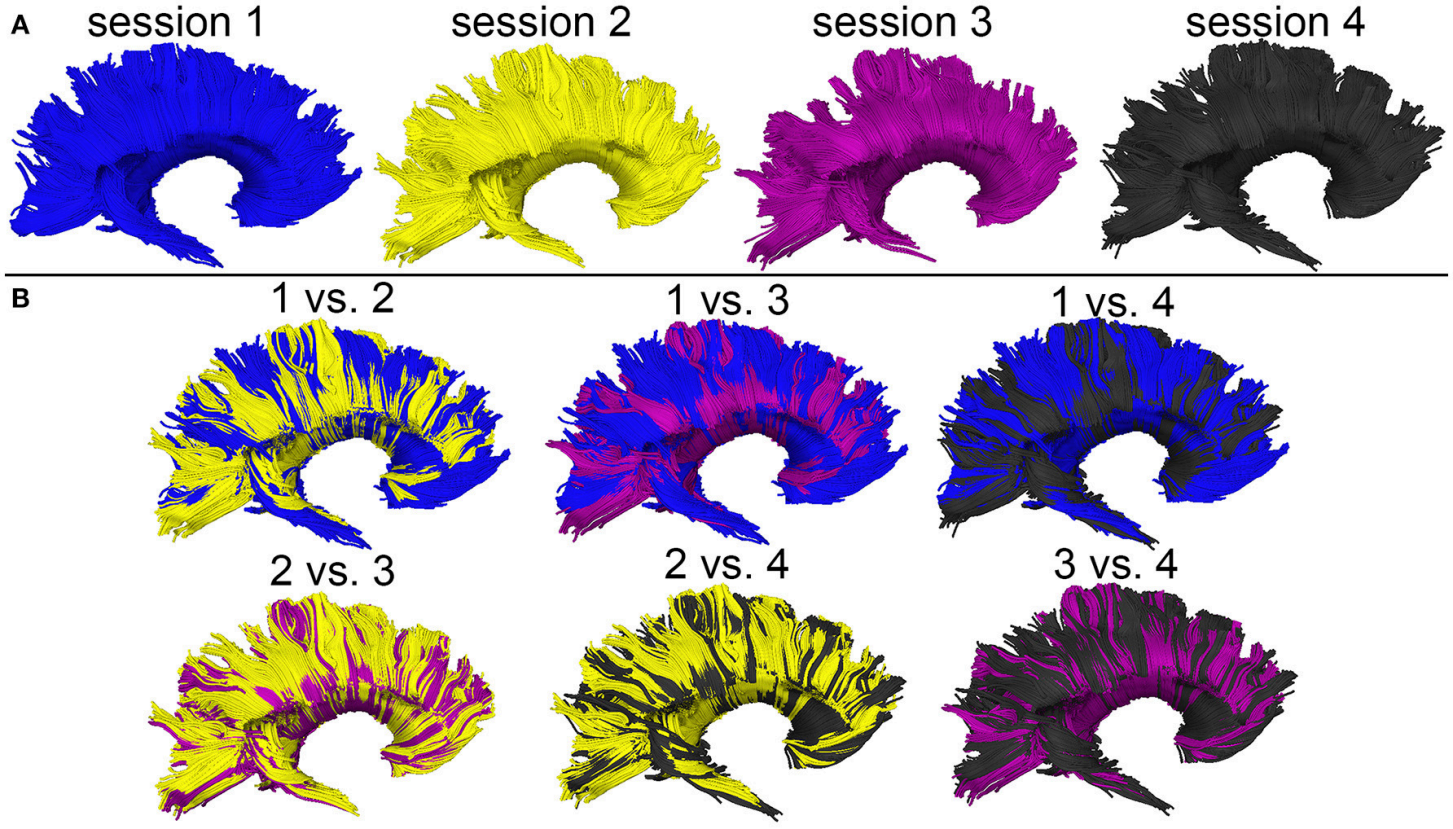
WM streamline reconstructions based on DWI volumes acquired during four distinct sessions scan session are displayed both separately **(A)** and in overlap with reconstructions from other sessions **(B)**. Careful visual examination and comparison of these reconstructions reveal differences which are likely the cumulative result of confounds such as motion artifacts, magnetic field inhomogeneity differences across scans, diffusion tensor estimation errors, etc.

Figure 3 shows the *T*_1_-weighted MRI scans of a younger HC adult whose DTI-derived callosal streamlines (as reconstructed based on scans from two distinct sessions held 1 week apart) are superimposed on anatomic MR images. The two DTI reconstructions (blue and yellow, Figure 3A) are similar, although noticeable differences are also visible. In this example, such differences are unlikely to be due to rigid co-registration errors alone because we were unable to explain them successfully using either manual co-registration or automatic co-alignment using a gradient-descent optimization of all registration parameters. Nevertheless, when displayed using red-green-blue (RGB) encoding of streamline orientations, the DTI streamlines derived from scans acquired during session 1 (Figure 3B) are consistent with satisfactory, adequate reconstructions of the corpus callosum, based on their agreement with the widely-mapped neuroanatomy of this structure (31). This conclusion emerges from the inspection of Figure 4 as well, where DTI reconstructions for each scan session are displayed both separately (Figure 4A) and in overlap with reconstructions from other sessions (Figure 4B). For the young HC volunteer whose callosal streamlines are shown in Figures 3, 4, the uncertainty radius is found to have μ = 2.7 mm and σ = 0.8 mm, with μ and σ being computed only over DWI voxels which exclusively contain callosal streamlines. In the other young HC volunteers, uncertainty radii are comparable (volunteer 2: μ = 2.9 mm, σ = 0.7 mm; volunteer 3: μ = 3.1 mm, σ = 0.4 mm; volunteer 4: μ = 2.8 mm, σ = 0.5 mm; volunteer 5: μ = 2.9 mm, σ = 0.9 mm; volunteer 6: μ = 2.7 mm, σ = 1.1 mm).

### Perilesional streamline matching

**Figure 5:**
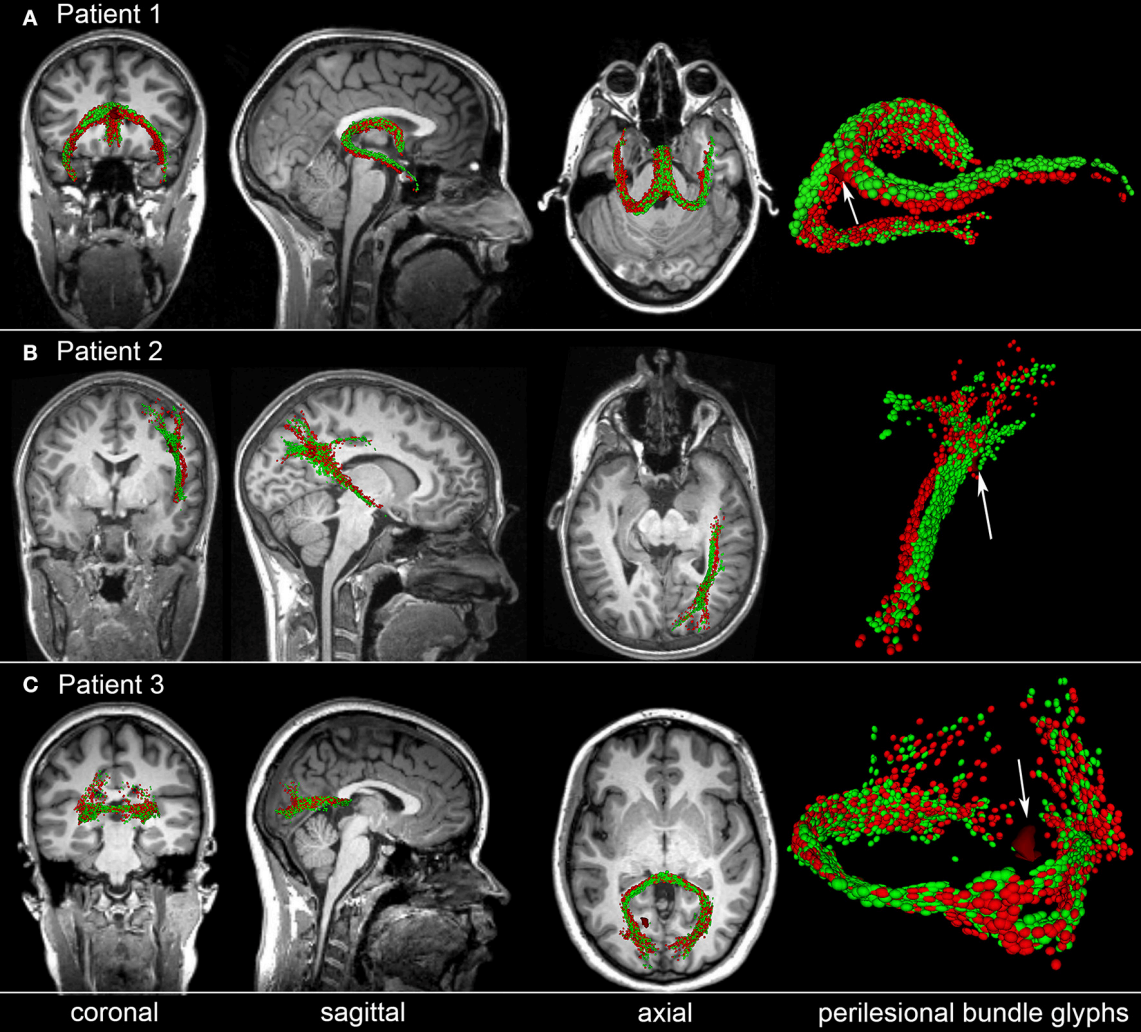
Representative examples of perilesional streamlines in three TBI victims. For each case, standard views (coronal, sagittal, axial) of *T*_1_-weighted MRI are shown in addition to DTI glyphs associated with perilesional WM streamline bundles imaged acutely (red) and chronically (green). White arrows indicate CMB locations. **(A)** Patient 1 exhibits a CMB near the fornix, near the hippocampal commissure. Six months after injury, the location and trajectory of the right fimbria are visibly different from their acute presentation. **(B)** Patient 2 has a CMB in the deep WM, near connections between the right parietal and temporal lobes; glyph comparison indicates a spatial shift of the longest streamline bundle toward the longitudinal fissure. **(C)** Patient 3 has a hemorrhage close to a left-hemisphere streamline bundle belonging to the splenium of the corpus callosum. The splenium is notably asymmetric at both time points, with the asymmetry being close to the CMB. If a causal relationship between the CMB and this neuroanatomic correlate could be established, this example would provide proof-of-concept that some CMB-related alterations in brain structure can persist for months after the traumatic event.

To illustrate the type of analysis which can be carried out using our streamline identification and matching approach, Figure 5 shows representative examples of perilesional streamlines in three mTBI victims. Patient 1 (Figure 5A) is a female mTBI victim with upper good recovery [indicated by a Glasgow Outcome Scale-Extended (GOS-E) of 8] who, acutely, was found to exhibit a ~4 mm^3^ CMB within the fornix, near the hippocampal commissure. At the chronic time point (~6 months after injury), the location and trajectory of the right fimbria are found to differ visibly compared to the acute time point. Patient 2 (Figure 5B) is a male mTBI victim (GCS = 13) with upper good recovery (GOS-E = 8) and with a ~3 mm^3^ CMB located near a DTI streamline bundle connecting the right parietal and temporal lobes. Comparison of the glyphs across time points suggests that, 6 months after injury, the bundle in question shifted slightly inwards and toward the right lateral ventricle, possibly due to decreased inflammation and/or intracranial pressure. Patient 3 (Figure 5C) is a male mTBI victim with a ~4 mm^3^ CMB close to a left-hemisphere streamline bundle belonging to the splenium of the corpus callosum. There is notable asymmetry of the depicted structure with respect to the longitudinal fissure, with brief—though clear—separation of the WM streamline trajectories ipsilateral to the CMB. Furthermore, the asymmetry is not found to have resolved by the time of the chronic scan. Together, these results suggest that CMBs can be present near WM streamlines which exhibit visible acute deformations and/or chronic trajectory changes even when CMB volume is relatively small. Furthermore, due to the asymmetry of the streamlines in Patient 3 and to the deformations observed ipsilateral to the lesion, the findings in this patient suggest that such WM changes may (occasionally) involve CMBs biomechanically and perhaps even causally. We propose that further analysis of CMBs in a larger patient sample is required for rigorous testing of these hypotheses.

### Along-tract analysis

In HC volunteers, as expected, the along-tract analysis failed to identify statistically-significant differences in the mean FA of any DTI streamline bundles. In the mTBI group, significant differences in the mean FA of peri-lesional streamline bundles were found in 21 of 26 volunteers. All these differences involved negative differences (decreases) in mean FA. In the 21 volunteers where significant differences had been found, an average of 47% of all identified TBI-related CMBs (σ = 21%) were in the spatial neighborhoods of DTI streamline bundles which exhibited statistically-significant (*p* < 0.001) differences in mean FA across time points. In other words, given an mTBI victim with TBI-related CMBs, the expected probability for any of that volunteer's CMBs to have neighboring (perilesional) streamline bundles with statistically-significant (*p* < 0.001) differences in mean FA across time was 47%. No immediately-discernable criterion could be identified to distinguish TBI-related CMBs associated with significant WM differences from those which were not. Further study of larger cohorts is likely necessary to identify the biological factors responsible for causing only certain perilesional WM streamlines to degrade over time.

## Discussion

### Background, motivation and significance

The advent of personalized medicine, coupled with growing interest in quantifying changes to cortical structure and/or WM circuitry prompted by gross pathology, have led to renewed efforts toward developing methods which can facilitate DWI/DTI analysis in longitudinal—rather than cross-sectional—studies, particularly studies of neurotrauma. Writing about the relative advantages and shortcomings of both approaches, Aarnink et al. (32) noted the benefits of automating intra-subject WM analysis across time points while implementing this operation separately for each subject in a cohort. Such an approach can allow researchers to reduce the adverse effects of inter-subject variability upon the reliability of statistical analysis, while preserving the efficiency and objectivity of automated quantification methods.

Many longitudinal DWI/DTI analysis methods—including voxel-based morphometry (VBM), tract-based spatial statistics (TBSS), automated longitudinal intra-subject analysis (ALISA) and similar approaches—use skeletons, atlases or similar constructs based on inter-subject averaging, whether for brain-wide or for structure-specific analysis. By contrast, to identify tract correspondences across time points in the presence of lesions (as in the case of TBI, stroke or MS), the current study advocates the use of a subject-specific approach whose aim is to implement a rigorous, longitudinal analysis of WM tracts located in the (pen)umbrae (spatial neighborhoods) of such lesions. One motivation behind this strategy is the considerable clinical interest in understanding how pathology-related changes in local brain structure, metabolism and blood oxygenation affect portions of brain tissue which are adjacent to pathology and which may appear healthy on MRI scans despite this not always being the case. For example, in mTBI patients who exhibit CMBs throughout the brain parenchyma, it is far from clear whether these forms of pathology are clinically silent or whether their presence can give rise to WM reorganization and to subsequent neurological and/or cognitive deficits (33). Though pathology-tailored MRI sequences such as fluid-attenuated inversion recovery (FLAIR) may be combined with DWI/DTI to obviate the presence of cytotoxic and vasogenic edema in CMB penumbrae, the effect of transient local inflammation upon WM circuitry is poorly understood and worthy of further investigation (34). Similarly, WM connections passing through brain regions directly affected by stroke or by MS lesions can change in ways which cannot be made plain by noninvasive imaging methods currently available. Nevertheless, injury-related mechanisms may lead to the reorganization of the connectome and to neurological impairments (35). For this reason, the availability of robust methods for the assessment of brain circuitry changes after neurotrauma is increasingly useful and desirable in the emerging era of personalized medicine.

### Comparison to VBM and TBSS

VBM is one of the earliest approaches for the longitudinal quantification of brain shape. When carefully applied and rigorously validated, VBM approaches can draw valid conclusions, even in the setting of a longitudinal analysis (36, 37). Nevertheless, one frequently-quoted disadvantage of this method is that, as originally proposed and implemented, it cannot typically resolve the ambiguity of whether apparent longitudinal differences in WM properties across time points are truly due to WM changes or rather to local mis-registration of DWI volumes (2). For example, it is typically unsafe to assume that even a nonlinear registration with many degrees of freedom can align DWI/DTI volumes well enough to permit the unambiguous interpretation of voxel-wise statistics. The approach of the present study performs co-registration of DTI streamlines rather than of DWI volumes, and our uncertainty radius quantifications highlight the drawbacks of using VMB for within-subject, longitudinal DTI analysis.

The widely-applied TBSS method was introduced to alleviate some of the perceived shortcomings of VBM (38). In TBSS, localized statistical testing of FA and other DWI-derived measures is used to reduce image misalignment by projecting maps of these measures into a common space. Specifically, after an initial, approximate nonlinear registration, TBSS projects the FA maps of individual subjects onto an alignment-invariant tract called the mean FA skeleton. Though allegedly superior to VBM in some ways, TBSS has its own limitations. For example, it has been argued that, as in VBM, longitudinal analyses of WM integrity using TBSS remain highly dependent upon the accurate registration of DWI/DTI volumes, which can be challenging to achieve in the presence of brain malformations or of mass effects which result in large anatomical shifts, like in TBI, stroke, and MS (1). Another limitation is that only those tracts which can be reliably traced and separated from others can be used to create a trustworthy FA parametrization; this shortcoming can sometimes make TBSS unamenable to the study of WM connectivity affected by mass effects and by their underlying pathology. In TBI, for example, structural abnormalities may be present far from the FA skeleton used in TBSS, such that it may not be possible to assess local pathology effects unless the lesions of interest are in immediate proximity to the WM tracts which form this skeleton. In our approach, longitudinal DTI analysis can be implemented for arbitrary locations within the WM, whether such locations are near or far from the TBSS skeleton. Furthermore, the tract-matching technique implemented here can ensure that only streamlines with consistent trajectories across time points are included in the analysis.

### Comparison to parametric surface methods

To overcome some of the limitations inherent to TBSS, Yushkevich et al. (39) argued in favor of taking into account the unique properties of specific anatomical structures and proposed that focusing on such structures is often more appropriate than performing analyses over the entire brain. These authors made the astute observation that an analysis which restricts its attention to structures of specific interest produces inferences which are also structure-specific, and which can be communicated and visualized more effectively than whole-brain results. Instead of using skeletonization (as in TBSS), Yushkevich et al. represented the skeletons of their structures of interest as parametric surfaces which facilitate manifold-based statistical analyses similar to those used on inflated cortical maps (40). In their approach, deformable medial models are fit to binary segmentations of fasciculi in a common space (atlas) to describe the skeleton and boundaries of a geometrical object as parametric surfaces with pre-defined topology. This method is most frequently applied to population-level studies, and rather rarely to within-subject studies. Nevertheless, its validity and potential advantages for segmentation, modeling and analysis in subject space—where shape analysis can be critical for identifying tracts across time points—have been pointed out (39), such that the method may constitute a useful alternative to our own.

Partially inspired by the work of Yushkevich et al. (39), Aarnink et al. (32) advocate the use of ALISA. In ALISA, DWI/DTI volumes acquired longitudinally from each participant are averaged over to create a subject-specific DWI/DTI template which is then applied at every time point, as necessary. One challenge of implementing this method, however, arises when not enough longitudinal scans are available to generate a subject-specific atlas which is sufficiently unsusceptible to noise, motion artifacts, etc. Another difficulty presents itself if brain structure changes substantially across time points, as in the case of typical development, neurodegeneration or in pathological conditions like those of interest here. These considerations set the stage and argue in favor of our own methodological approach, whose most prominent features are justified and discussed in what follows.

### Longitudinal analysis via lesion-robust curve matching

Performing tractography at each time point within a given subject and then matching the resulting streamlines across time points stands in contrast to the scenario where DWI volumes are first co-registered and then used to perform tractography. In our case, part of the rationale for adopting the former—rather than the latter—strategy is the fact that, due to pathology evolution, longitudinal imaging datasets containing lesions may exhibit substantial topology changes across time. This phenomenon and related challenges may result in localized deformations of WM tracts, causing substantial local errors in volume co-registration. In turn, this can lead to the reconstruction of tractography streamlines whose similarity is artificially imposed by the volume co-registration process, rather than by their true geometric resemblance. By generating tractography streamlines at each time point and then matching streamlines across time points, more insight into true tract shape changes across time can be gained without the potential confound of DWI/DTI volume co-registration prior to tractography.

### Topology-informed curve matching

Of substantial significance during longitudinal DWI/DTI analysis is the method used for matching streamline bundles; in the present study, this is particularly important when matching prototyped streamlines. In DTI, the assumption of curve index correspondence between streamlines imaged at different time points may conceivably hold for cases where there are small, diffeomorphic changes in space curve properties across time points. This assumption, however, can be easily violated in cases where gross neuropathology causes substantial curve deformation. For example, the spatial expansion of a hemorrhagic lesion often leads to the shearing and tearing of axons, which may raise challenges when attempting to identify WM fiber portions which exhibit injury-related alterations. For these and other reasons, it is important to reduce the confounding effects of both spatial distortions as well as DWI/DTI measurement variability across time points when implementing curve index correspondence. A powerful approach to this problem, as proposed by O'Donnell et al. (41), involves taking advantage of WM bundles being relatively symmetric with respect to the longitudinal fissure, such that generating arc length correspondences across hemispheres can be used to enforce appropriate curve index correspondence. Whereas this approach can indeed be very useful when studying either healthy brains or brains affected by disorders which are expected to affect both hemispheres equally, invoking symmetry arguments in cases such as ours can be nefarious for at least three important reasons. Firstly, the asymmetric nature of brain lesion sizes and distributions implies that the mirror symmetry of WM tracts in the two hemispheres of the brain may not be preserved after injury. Secondly, both local and global edema may affect WM tract trajectories, thereby creating or augmenting inter-hemispheric asymmetry. Thirdly, even in the absence of lesion-related effects, the phenomenon of lateralization and its underlying structural basis can preclude the existence of inter-hemispheric arc length correspondence in cases where substantial inter-subject variability exists. For all these reasons, though our work is inspired by that of O'Donnell and Westin (26), we advocate identifying curve index correspondences without resorting to brain symmetry assumptions.

### Curve index correspondence

Various approaches have been used to implement curve index correspondence, a very successful one being the prototype streamline calculation method (42, 43), where a prototype streamline that is representative of an entire bundle is identified. In applications such as ours—where along-streamline statistics for WM tracts in (pen)umbral areas are of clinical interest—the ability to calculate prototype streamlines is of key importance for several reasons. Firstly, lesions may occur anywhere in the brain, e.g., at locations where both major and minor tracts intersect, such that relying on tractography results in the absence of streamline prototyping can lead to substantial error. This is because perilesional streamlines (1) may belong to more than one major fasciculus or bundle, and (2) can have spatial trajectories ranging from largely-uniform to highly-diffuse. In such cases—particularly in situations where the tracts of interest have highly-variable trajectories—choosing prototype streamlines along each of these trajectories can be important when attempting to alleviate the potential effects of noise, subject motion, structural brain changes and of other factors which may negatively impact tractography or subsequent along-tract analysis.

### Tract parametrization approaches

The practice of parametrizing WM tracts to find trajectory correspondences can be implemented in various ways, e.g., (1) using manual specification of fiducial points along streamlines by a human expert (44), (2) by assuming endpoint correspondences to align curves (42), (3) via implementation of statistical bundle models with point correspondences to perform streamline clustering and measurement (27), (4) based on skeletons to define locations which correspond to the central portions of bundles like in TBSS (2), or (5) using the tractography-derived medial model of Yushkevich et al. (39). More recently, O'Donnell et al. (41) proposed calculating mean bundle trajectories and various diffusion-related metrics in the coordinate system of prototype bundles so as to facilitate along-tract statistical comparisons across subjects using TBM. In contrast to TBSS and to medial model approaches, TBM employs subject-specific tractography segmentations instead of relying on an atlas, which is an important commonality between TBM and the present approach.

### DTI analysis in the spatial neighborhood of lesions

Besl and McKay (45) introduced an influential, iterative closest point (ICP) algorithm for the co-registration of three-dimensional (3D) parametric space curves defined as linear combinations of cubic B-splines and control points. Partially inspired by this approach, Leemans et al. (24) proposed an automatic, multi-scale, feature-based, rigid-body co-registration technique for DTI tractography bundles. In the latter method, minimization of the mean-squared difference between corresponding tract pathways in the parameter space formed by tract curvature and torsion is attempted. Importantly, this technique is adequate for local transformations within regions of interest (ROIs). In ALISA, Aarnink et al. (32) define a single ROI using a subject-specific template, after which the same ROI is applied to the DWI/DTI volumes acquired at each time point. This approach, however, assumes negligible geometric variations across time points, and this assumption is often false when lesions are present. Because of this, accounting for changes in lesion size and shape can be essential when attempting to quantify the effects of brain injury upon WM connectivity. To accommodate these requirements, our present approach relies on delineating the (pen)umbrae of lesions as ROIs within each subject. The DTI streamline segments within each ROI are then identified across time points using the results of curve index correspondence calculations to identify the streamline portions to which the segments in question belong.

### Fiber selection for along-tract analysis

In our case, the validity of along-tract analysis is highly reliant upon the ability to perform fiber matching and tract prototype selection. To select prototype DTI tractography bundles, one can alternatively choose (1) the longest streamline in the bundle of interest, (2) the longest streamline weighted by some local measure of streamline integrity, or (3) the streamline which is most representative of the bundle trajectory according to some appropriate measure, such as the O'Donnell-Westin streamline affinity metric (26). In applications such as ours, the first two methods can result in poor streamline prototyping because some streamlines may be elongated by gross pathology processes, and this can negatively affect the identification of appropriate curve index correspondences. Choosing the longest streamline available can negatively impact this process as well because maximum streamline length is a metric vulnerable to DWI measurement noise, to DTI tractography artifacts and to other sources of error which can lead to both global and local instability while solving the optimization problems associated with curve index correspondence calculations or with fiber prototype selection. For these reasons, O'Donnell's streamline affinity approach appears to be more suitable for applications such as ours, due to its greater stability compared to the other two methods. When prototype streamline selection is implemented for sets of streamlines whose spatial trajectories are (highly) variable, however, the number of prototype streamlines identified by the method may be larger than typically expected. In such cases, an important and interesting observation on streamline prototyping in our approach is that performing fiber selection is somewhat analogous to the application of a low-pass, three-dimensional spatial filter to DWI/DTI image intensities with the goal of identifying the most significant and most topologically-consistent directions of water diffusion within a ROI. However, in contrast to 3D smoothing of DWI/DTI volumes (which is topology-agnostic), the prototyping procedure advocated here takes into consideration the topology of WM streamline trajectories, their fundamental properties (κ and τ) as well as the underlying neuroanatomy of the brain, all of which can result in a relatively more principled way of attenuating the effects of measurement noise, motion artifacts and of other confounds.

### Implications and conclusion

The task of longitudinal, within-subject analysis of WM changes in the human brain is of substantial interest in studies of aging, brain injury, dementia, development, etc. Although strategies for population-level studies have received comparably more attention than methods for single-subject analysis, the advent of personalized medicine and the heterogeneity of brain injury patterns indicate that the need for techniques which can accommodate single-subject analysis and profiling is likely to increase in the foreseeable future. Such analysis can be greatly complicated by the presence of both hemorrhagic and non-hemorrhagic lesions, and the effects of such lesions upon WM circuitry has not been studied sufficiently. Nevertheless, understanding the effect of blood-brain barrier breakdown due to hemorrhagic TBI is likely important because CMBs occur in a sizeable number of TBI victims, including mTBI (3). The analysis workflow demonstrated in this study allows one to identify CMBs automatically, to match perilesional DTI streamline bundles across scans in principled ways, and to identify WM structures whose properties differ significantly across time points in victims of neurological disease compared to age- and sex-matched HC volunteers. As illustrated here, longitudinal DTI analysis can be confounded both by MR measurement artifacts and by technical limitations, to the extent that methods which are neuroanatomy-agnostic may be unreliable. Partly for this reason, longitudinal analysis techniques which incorporate constraints pertaining to local WM structure (e.g., streamline prototyping and matching) are likely to provide more realistic information than topology-agnostic approaches. Furthermore, the ability to interpret WM differences across scans while also accounting for the expected variability of such differences in the presence of confounds is essential when developing principled strategies for the study of brain connectivity in both health and disease.

## Author contributions

KR and AM analyzed data, interpreted results, and wrote sections of the manuscript. AI designed the study, acquired imaging data, interpreted results, wrote sections of the manuscript and coordinated the study. All authors contributed to manuscript revision, read and approved the submitted version.

### Conflict of interest statement

The authors declare that the research was conducted in the absence of any commercial or financial relationships that could be construed as a potential conflict of interest.
